# Correction to: Metabolically-versatile *Ca*. Thiodiazotropha symbionts of the deep-sea lucinid clam *Lucinoma kazani* have the genetic potential to fix nitrogen

**DOI:** 10.1093/ismeco/ycae118

**Published:** 2025-06-23

This is a correction to: Lina Ratinskaia, Stas Malavin, Tal Zvi-Kedem, Simina Vintila, Manuel Kleiner, Maxim Rubin-Blum, Metabolically-versatile *Ca*. Thiodiazotropha symbionts of the deep-sea lucinid clam *Lucinoma kazani* have the genetic potential to fix nitrogen, *ISME Communications*, Volume 4, Issue 1, January 2024, https://doi.org/10.1093/ismeco/ycae076

In the originally published version of the manuscript, there was an error in the naming of the symbionts in the last paragraph of the Introduction section. In the following sentence, “*Ca*. Tuxoctenus gloverae” has been corrected to read “*Ca*. Thiodiazotropha gloverae”:

“The latter was described in the North Atlantic at depths down to 165 m and consistently hosts *Ca*. Tuxoctenus gloverae symbionts [13,24].𠇜

The corrected sentence reads as follows:

“The latter was described in the North Atlantic at depths down to 165 m and consistently hosts *Ca*. Thiodiazotropha gloverae symbionts [13,24].”

Section title “*Ca*. *T. Gloverae* Ex L. kazani are metabolically versatile” has been corrected to read “*Ca*. T. gloverae ex *L. kazani* are metabolically versatile”.

Section title “Potential features responsible for biotic interactions of *Ca*. T. *Gloverae* Ex. L. Kazani” has been corrected to read “Potential features responsible for biotic interactions of *Ca*. T. gloverae ex *L. kazani*”.

In the following sentences, in the Abstract and the Introduction sections, the first mention of “*Ca*.” abbreviation has been expanded to “*Candidatus*”:

“Lucinids harbor *Candidatus* Thiodiazotropha symbionts that can oxidize inorganic and organic substrates such as hydrogen sulfide and formate to gain energy.”

“Lucinids are characterized by their obligate association with intracellular chemosynthetic bacteria, most often with *Candidatus* Thiodiazotropha (Chromatiales, Sedimaenticolaceae), and rarely with other gammaproteobacteria, such as Thiohalomonadales species [12,13].”

