# Supplementary material for: Metabolically-versatile Ca. Thiodiazotropha symbionts of the deep-sea lucinid clam Lucinoma kazani have the genetic potential to fix nitrogen
Source: ISME Commun. 2024 Jun 4;4(1):ycae076. doi: 10.1093/ismeco/ycae076 (PMC11171427; doi:10.1093/ismeco/ycae076)
Supplement: Supplementary_Material_ycae076 [file supplementary_material_ycae076.pdf]

Supplementary Material

Metabolically-versatile *Ca.* Thiodiazotropha symbionts of the deep-sea lucinid clam *Lucinoma kazani* have the genetic potential to fix nitrogen

Lina Ratinskaia, Stas Malavin, Tal Zvi-Kedem, Simina Vintila, Manuel Kleiner, Maxim Rubin-Blum

Supplementary Note 1

Prolonged sample retrieval and ex-situ fixation may alter symbiont gene expression, given the changes in environmental conditions, which impose stress on the host and the symbionts. Oxygen deprivation is a likely stressor during retrieval because valves are closed and gills are not ventilated. but also shifts in temperature and availability of dissolved gases, among others. For example, stress- and denitrification-related genes were upregulated in symbionts of bathymodioline mussels that were fixed onboard, compared to in situ fixation [1]. In our study, proteomics shows that the abundance of stress-related proteins, such as chaperone protein ClpB and heat shock protein GrpE may positively correlate with that of proteins involved in denitrification (NapA, NarG, NosZ) (**Supplementary Figure S4**). In turn, the abundance of RbcL (form I RubisCo large subunit), but not that of CbbM (form II RubisCo), appears to decrease, as the abundance of stress proteins increases (**Supplementary Figure S4**). Both processes are likely triggered by oxygen deprivation.

1. Tietjen M. Physiology and ecology of deep-sea *Bathymodiolus* symbioses. 2020. Universität Bremen.

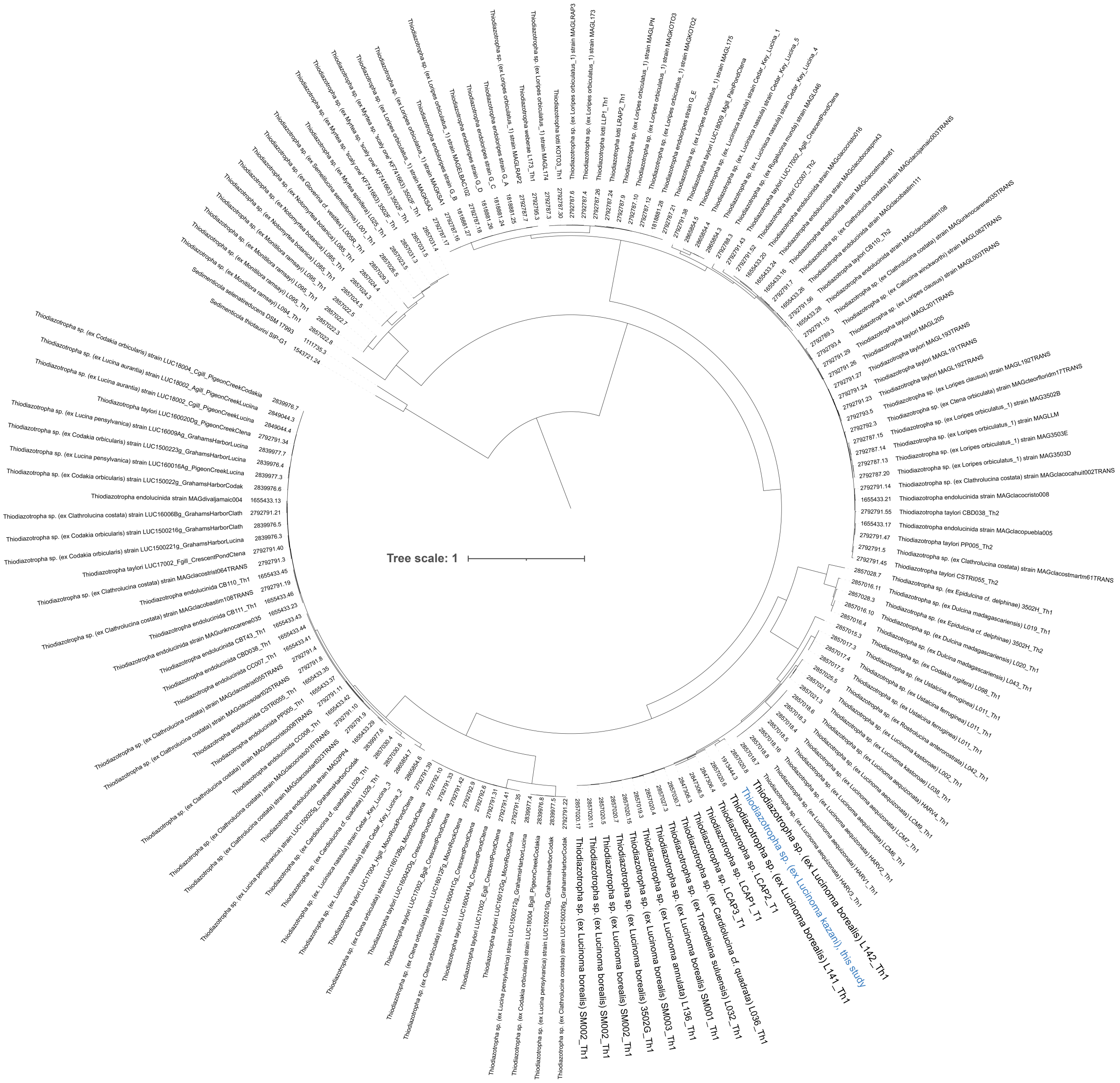

Supplementary Figure S1: Phylogenomic tree of 150 genomes in the Thiodiazotropha clade (2 *Sedimenticola* genomes were added for an outgroup). The tree is based on 411 single-copy genes and uses the JTT model in RAxML. The tree is drawn to scale and rooted at the midpoint. Numbers represent BV-BRC accessions.

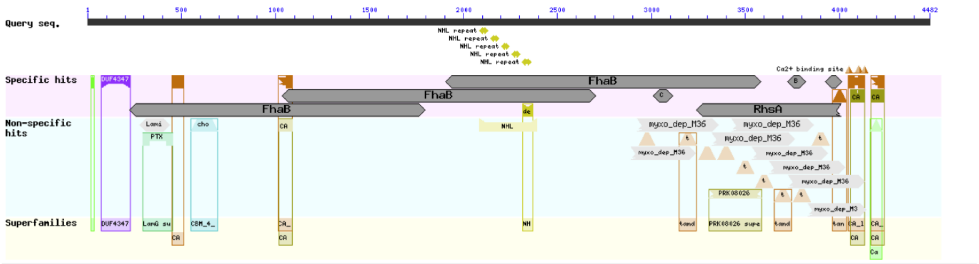

Search for similar domain architectures | Refine search

List of domain hits

Name	Accession	Description	Interval	E-value
[+] DUF4347	pfam14252	Domain of unknown function (DUF4347); This domain family is found in bacteria and eukaryotes, ...	74-229	1.22e-56
[+] FhaB	COG3210	Large exoprotein involved in heme utilization or adhesion [Intracellular trafficking, ...	1907-3582	3.83e-40
[+] FhaB	COG3210	Large exoprotein involved in heme utilization or adhesion [Intracellular trafficking, ...	1034-2705	3.80e-29
[+] Cadherin_3	pfam16184	Cadherin-like.	3009-3113	6.09e-28
[+] FhaB	COG3210	Large exoprotein involved in heme utilization or adhesion [Intracellular trafficking, ...	224-1798	4.66e-21
[+] myxo_dep_M36	NF038112	myxosortase-dependent M36 family metallopeptidase. Members of this bacterial protein family ...	3428-3864	5.37e-21
[+] myxo_dep_M36	NF038112	myxosortase-dependent M36 family metallopeptidase. Members of this bacterial protein family ...	3325-3763	1.70e-20
[+] myxo_dep_M36	NF038112	myxosortase-dependent M36 family metallopeptidase. Members of this bacterial protein family ...	3538-3941	1.51e-19
[+] myxo_dep_M36	NF038112	myxosortase-dependent M36 family metallopeptidase. Members of this bacterial protein family ...	3628-4033	1.16e-18
[+] myxo_dep_M36	NF038112	myxosortase-dependent M36 family metallopeptidase. Members of this bacterial protein family ...	3729-4136	6.12e-17
[+] delta_60_rpt	TIGR02608	delta-60 repeat domain; This domain occurs in tandem repeats, as many as 13, in proteins from ...	2316-2369	7.20e-16
[+] myxo_dep_M36	NF038112	myxosortase-dependent M36 family metallopeptidase. Members of this bacterial protein family ...	2928-3359	1.03e-15
[+] Big_9	pfam17963	Bacterial Ig domain. This entry represents a wide variety of bacterial Ig domains.	3928-4015	1.45e-14
[+] tand_rpt_95	NF012211	tandem-95 repeat; This 95-amino acid repeat occurs in tandem in proteins that may be several ...	3963-4039	3.75e-13
[+] tand_rpt_95	NF012211	tandem-95 repeat; This 95-amino acid repeat occurs in tandem in proteins that may be several ...	3655-3746	2.27e-12
[+] tand_rpt_95	NF012211	tandem-95 repeat; This 95-amino acid repeat occurs in tandem in proteins that may be several ...	3150-3241	2.36e-12
[+] tand_rpt_95	NF012211	tandem-95 repeat; This 95-amino acid repeat occurs in tandem in proteins that may be several ...	3352-3443	3.10e-12
[+] tand_rpt_95	NF012211	tandem-95 repeat; This 95-amino acid repeat occurs in tandem in proteins that may be several ...	3453-3544	3.62e-12
[+] Big_9	pfam17963	Bacterial Ig domain. This entry represents a wide variety of bacterial Ig domains.	3726-3822	1.18e-11
[+] tand_rpt_95	NF012211	tandem-95 repeat; This 95-amino acid repeat occurs in tandem in proteins that may be several ...	3251-3342	1.97e-11
[+] tand_rpt_95	NF012211	tandem-95 repeat; This 95-amino acid repeat occurs in tandem in proteins that may be several ...	3554-3645	5.27e-11
[+] tand_rpt_95	NF012211	tandem-95 repeat; This 95-amino acid repeat occurs in tandem in proteins that may be several ...	3756-3847	6.04e-11
[+] Cadherin_repeat	cd11304	Cadherin tandem repeat domain; Cadherins are glycoproteins involved in Ca2+-mediated cell-cell ...	4048-4137	1.01e-10
[+] myxo_dep_M36	NF038112	myxosortase-dependent M36 family metallopeptidase. Members of this bacterial protein family ...	2891-3235	2.24e-10
[+] NHL	cd05819	NHL repeat unit of beta-propeller proteins; The NHL(NCL-1, HT2A and LIN-41)-repeat is found in ...	2087-2392	8.71e-10
[+] tand_rpt_95	NF012211	tandem-95 repeat; This 95-amino acid repeat occurs in tandem in proteins that may be several ...	3857-3947	3.15e-09
[+] Laminin_G_3	pfam13385	Concanavalin A-like lectin/glucanases superfamily. This domain belongs to the Concanavalin ...	279-437	5.67e-09
[+] RhsA	COG3209	Uncharacterized conserved protein RhaS, contains 28 RHS repeats [General function prediction ...	3240-4012	1.30e-07
[+] Cadherin_repeat	cd11304	Cadherin tandem repeat domain; Cadherins are glycoproteins involved in Ca2+-mediated cell-cell ...	4169-4242	2.99e-07
[+] CA	smart00112	Cadherin repeats; Cadherins are glycoproteins involved in Ca2+-mediated cell-cell adhesion. ...	4060-4137	2.16e-06
[+] myxo_dep_M36	NF038112	myxosortase-dependent M36 family metallopeptidase. Members of this bacterial protein family ...	3836-4143	3.43e-06
[+] tand_rpt_95	NF012211	tandem-95 repeat; This 95-amino acid repeat occurs in tandem in proteins that may be several ...	2933-3019	3.88e-06
[+] Cadherin_repeat	cd11304	Cadherin tandem repeat domain; Cadherins are glycoproteins involved in Ca2+-mediated cell-cell ...	1014-1090	1.23e-05
[+] CA	smart00112	Cadherin repeats; Cadherins are glycoproteins involved in Ca2+-mediated cell-cell adhesion. ...	4169-4242	1.89e-05
[+] Cadherin_repeat	cd11304	Cadherin tandem repeat domain; Cadherins are glycoproteins involved in Ca2+-mediated cell-cell ...	450-510	2.01e-05
[+] CA	smart00112	Cadherin repeats; Cadherins are glycoproteins involved in Ca2+-mediated cell-cell adhesion. ...	1016-1090	8.70e-04
[+] Cadherin	pfam00028	Cadherin domain;	4163-4229	1.50e-03
[+] PRK08026	PRK08026	FliC/FljB family flagellin;	3307-3588	2.25e-03
[+] choice_anch_C	TIGR04362	choice-of-anchor C domain; This family describes an extracellular bacterial domain that occurs ...	550-690	2.29e-03
[+] LEPR-8K	NF012209	LEPR-XLL family repeat protein signature domain; This model, just 24 amino acids long, ...	16-36	4.29e-03
[+] PTX	smart00159	Pentraxin / C-reactive protein / pentaxin family; This family form a doscoid pentameric ...	296-453	9.30e-03

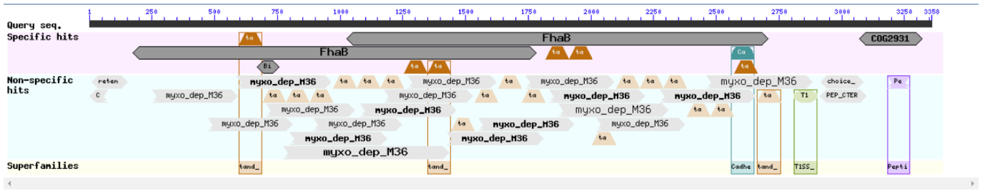

Search for similar domain architectures | Refine search

List of domain hits

Name	Accession	Description	Interval	E-value
[+] FhaB	COG3210	Large exoprotein involved in heme utilization or adhesion [Intracellular trafficking, ...	1026-2708	2.22e-35
[+] FhaB	COG3210	Large exoprotein involved in heme utilization or adhesion [Intracellular trafficking, ...	172-1782	7.59e-30
[+] myxo_dep_M36	NF038112	myxosortase-dependent M36 family metallopeptidase. Members of this bacterial protein family ...	1270-1622	1.46e-25
[+] myxo_dep_M36	NF038112	myxosortase-dependent M36 family metallopeptidase. Members of this bacterial protein family ...	1740-2092	5.80e-23
[+] myxo_dep_M36	NF038112	myxosortase-dependent M36 family metallopeptidase. Members of this bacterial protein family ...	1834-2214	1.09e-22
[+] retention_LapA	NF033682	retention module-containing protein. The retention module, as described for the giant adhesin ...	11-153	2.55e-21
[+] myxo_dep_M36	NF038112	myxosortase-dependent M36 family metallopeptidase. Members of this bacterial protein family ...	1176-1528	3.35e-21
[+] myxo_dep_M36	NF038112	myxosortase-dependent M36 family metallopeptidase. Members of this bacterial protein family ...	2462-2881	2.07e-19
[+] myxo_dep_M36	NF038112	myxosortase-dependent M36 family metallopeptidase. Members of this bacterial protein family ...	2277-2656	4.58e-18
[+] tand_rpt_95	NF012211	tandem-95 repeat; This 95-amino acid repeat occurs in tandem in proteins that may be several ...	597-689	5.53e-18
[+] Cadherin_5	pfam17892	Cadherin-like domain;	2559-2649	7.33e-18
[+] tand_rpt_95	NF012211	tandem-95 repeat; This 95-amino acid repeat occurs in tandem in proteins that may be several ...	1349-1441	2.14e-17
[+] myxo_dep_M36	NF038112	myxosortase-dependent M36 family metallopeptidase. Members of this bacterial protein family ...	1082-1462	2.30e-17
[+] tand_rpt_95	NF012211	tandem-95 repeat; This 95-amino acid repeat occurs in tandem in proteins that may be several ...	2571-2663	3.38e-17
[+] tand_rpt_95	NF012211	tandem-95 repeat; This 95-amino acid repeat occurs in tandem in proteins that may be several ...	1819-1911	7.73e-17
[+] tand_rpt_95	NF012211	tandem-95 repeat; This 95-amino acid repeat occurs in tandem in proteins that may be several ...	1255-1347	4.20e-16
[+] tand_rpt_95	NF012211	tandem-95 repeat; This 95-amino acid repeat occurs in tandem in proteins that may be several ...	1913-2005	8.55e-16
[+] myxo_dep_M36	NF038112	myxosortase-dependent M36 family metallopeptidase. Members of this bacterial protein family ...	1873-2308	8.87e-16
[+] Big_9	pfam17963	Bacterial Ig domain. This entry represents a wide variety of bacterial Ig domains.	668-758	9.94e-16
[+] myxo_dep_M36	NF038112	myxosortase-dependent M36 family metallopeptidase. Members of this bacterial protein family ...	586-964	2.16e-15
[+] myxo_dep_M36	NF038112	myxosortase-dependent M36 family metallopeptidase. Members of this bacterial protein family ...	1552-1932	1.15e-14
[+] myxo_dep_M36	NF038112	myxosortase-dependent M36 family metallopeptidase. Members of this bacterial protein family ...	2022-2374	2.59e-14
[+] myxo_dep_M36	NF038112	myxosortase-dependent M36 family metallopeptidase. Members of this bacterial protein family ...	1428-1810	6.64e-14
[+] tand_rpt_95	NF012211	tandem-95 repeat; This 95-amino acid repeat occurs in tandem in proteins that may be several ...	878-971	2.90e-12
[+] tand_rpt_95	NF012211	tandem-95 repeat; This 95-amino acid repeat occurs in tandem in proteins that may be several ...	2101-2193	5.45e-12
[+] tand_rpt_95	NF012211	tandem-95 repeat; This 95-amino acid repeat occurs in tandem in proteins that may be several ...	1537-1629	9.41e-12
[+] tand_rpt_95	NF012211	tandem-95 repeat; This 95-amino acid repeat occurs in tandem in proteins that may be several ...	2006-2099	1.05e-11
[+] tand_rpt_95	NF012211	tandem-95 repeat; This 95-amino acid repeat occurs in tandem in proteins that may be several ...	2477-2569	1.99e-11
[+] tand_rpt_95	NF012211	tandem-95 repeat; This 95-amino acid repeat occurs in tandem in proteins that may be several ...	1066-1159	2.22e-11
[+] tand_rpt_95	NF012211	tandem-95 repeat; This 95-amino acid repeat occurs in tandem in proteins that may be several ...	691-783	2.42e-11
[+] tand_rpt_95	NF012211	tandem-95 repeat; This 95-amino acid repeat occurs in tandem in proteins that may be several ...	2289-2381	2.69e-11
[+] myxo_dep_M36	NF038112	myxosortase-dependent M36 family metallopeptidase. Members of this bacterial protein family ...	706-1059	3.03e-11
[+] COG2931	COG2931	Ca2+-binding protein, RTX toxin-related [Secondary metabolites biosynthesis, transport and ...	3072-3320	3.27e-11
[+] tand_rpt_95	NF012211	tandem-95 repeat; This 95-amino acid repeat occurs in tandem in proteins that may be several ...	2383-2475	3.57e-11
[+] tand_rpt_95	NF012211	tandem-95 repeat; This 95-amino acid repeat occurs in tandem in proteins that may be several ...	1443-1535	4.02e-11
[+] tand_rpt_95	NF012211	tandem-95 repeat; This 95-amino acid repeat occurs in tandem in proteins that may be several ...	2195-2287	6.10e-11
[+] tand_rpt_95	NF012211	tandem-95 repeat; This 95-amino acid repeat occurs in tandem in proteins that may be several ...	1725-1817	6.22e-11
[+] tand_rpt_95	NF012211	tandem-95 repeat; This 95-amino acid repeat occurs in tandem in proteins that may be several ...	1631-1723	9.01e-11
[+] tand_rpt_95	NF012211	tandem-95 repeat; This 95-amino acid repeat occurs in tandem in proteins that may be several ...	785-877	2.34e-10
[+] tand_rpt_95	NF012211	tandem-95 repeat; This 95-amino acid repeat occurs in tandem in proteins that may be several ...	1161-1253	2.82e-10
[+] tand_rpt_95	NF012211	tandem-95 repeat; This 95-amino acid repeat occurs in tandem in proteins that may be several ...	973-1065	6.77e-10
[+] myxo_dep_M36	NF038112	myxosortase-dependent M36 family metallopeptidase. Members of this bacterial protein family ...	894-1246	8.03e-10
[+] tand_rpt_95	NF012211	tandem-95 repeat; This 95-amino acid repeat occurs in tandem in proteins that may be several ...	2664-2757	1.37e-09
[+] myxo_dep_M36	NF038112	myxosortase-dependent M36 family metallopeptidase. Members of this bacterial protein family ...	473-810	3.19e-09
[+] myxo_dep_M36	NF038112	myxosortase-dependent M36 family metallopeptidase. Members of this bacterial protein family ...	774-1434	4.50e-09
[+] myxo_dep_M36	NF038112	myxosortase-dependent M36 family metallopeptidase. Members of this bacterial protein family ...	800-1186	7.34e-08
[+] COG4254	COG4254	Uncharacterized peptidoglycan binding protein, contains LysM and FecR domains [General ...	1-72	1.19e-07
[+] choice_anch_K	NF038131	choice-of-anchor K domain. The choice-of-anchor K is a rather uncommon extracellular domain ...	2918-3087	1.59e-07
[+] PEP_CTERM_THxN	NF038125	THxN family PEP-CTERM protein. This family of PEP-CTERM proteins is named for one of several ...	2913-3098	7.18e-07
[+] T1SS_rpt_143	TIGR03660	T1SS-143 repeat domain; This model represents a domain of about 143 amino acids that may occur ...	2809-2902	2.19e-06
[+] Peptidase_M10_C	pfam08548	Peptidase M10 serralysin C terminal; Serralysins are peptidases related to mammalian matrix ...	3183-3271	3.75e-06
[+] myxo_dep_M36	NF038112	myxosortase-dependent M36 family metallopeptidase. Members of this bacterial protein family ...	252-590	1.63e-04

Supplementary Figure S2: Domains of the two large toxin-like proteins in *Lucinoma kazani* genomes (CD-search, NCBI).

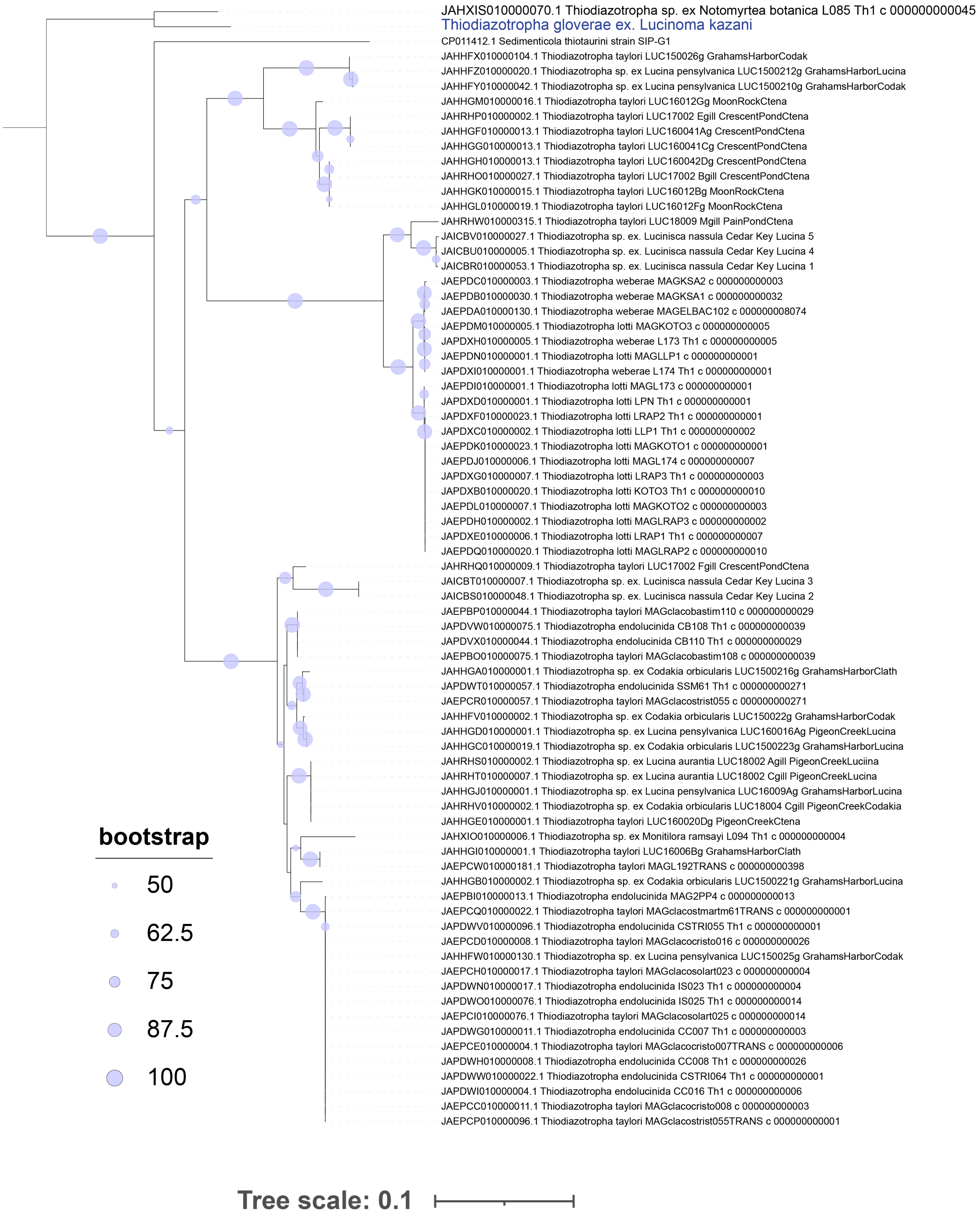

Supplementary Figure S3: Phylogeny of 73 nucleotide sequences encoding NifD in the Thiodiazotropha clade. The maximum likelihood tree is constructed in IQtree using the TN+F+I+G4 model. The tree is rooted at the midpoint. The tree scale represents the number of substitutions per site.

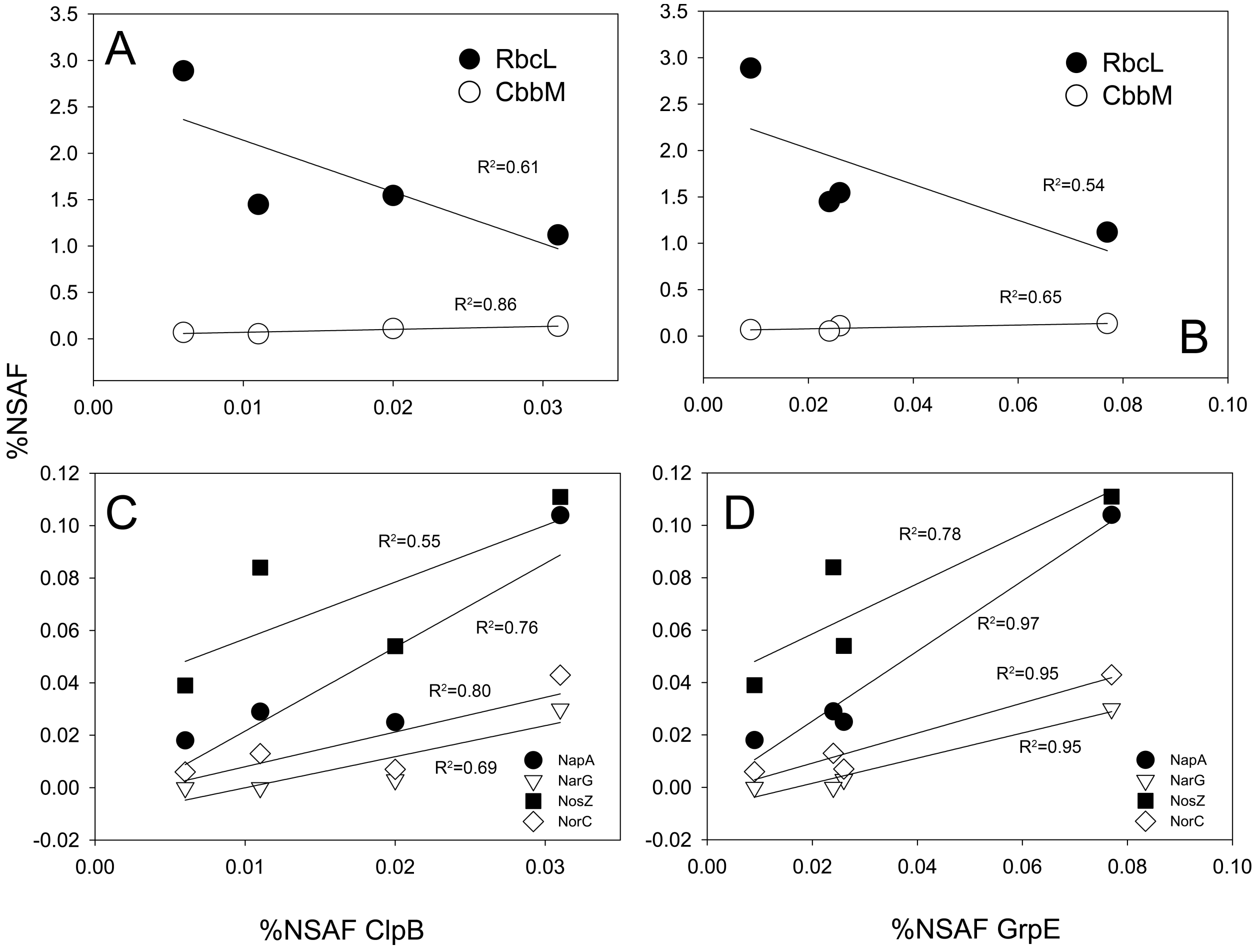

Supplementary Figure S4: The fraction of protein number of spectral counts (%NSAF) of stress-induced chaperone protein ClpB and heat shock protein GrpE is negatively correlated with that of RubisCO form I (RbcL large subunit; panels A and B), and positively correlated with %NSAF of RubisCO form II (CbbM; panels A and B), as well as with denitrification proteins (panels C, D).

Supplementary Table Legends:

Supplementary Table ST1: Stable isotope analyses of carbon, nitrogen and sulfur in *Lucinoma kazani* tissues.

Supplementary Table ST2: Genomic features of *Ca.* Thiodoazotropha ex. *Lucinoma kazani*. Expression values as RNA read counts and protein abundance as % normalized spectral abundance factor (NSAF) in gills samples of four *L. kazani* specimens. Red fonts represent annotations based on homology with NCBI/UniProt databases (defined as hypotheticals by RAST-tk annotation). Green cells represent putative prophage regions.

Supplementary Table ST3: Protein annotations for Figure 2 in the main text.
